# Dapagliflozin, SGLT2 Inhibitor, Attenuates Renal Ischemia-Reperfusion Injury

**DOI:** 10.1371/journal.pone.0158810

**Published:** 2016-07-08

**Authors:** Yoon-Kyung Chang, Hyunsu Choi, Jin Young Jeong, Ki-Ryang Na, Kang Wook Lee, Beom Jin Lim, Dae Eun Choi

**Affiliations:** 1 Department of Nephrology, Daejeon St. Mary Hospital, Daejeon, South Korea; 2 Department of Nephrology, Catholic University of Korea, Seoul, South Korea; 3 Clinical Research Institute, Daejeon St. Mary Hospital, Daejeon, South Korea; 4 Department of Nephrology, School of medicine, Chungnam National University, Daejeon, South Korea; 5 Department of Medical Science, School of medicine, Chungnam National University, Daejeon, South Korea; 6 Department of pathology, College of medicine, Yeonse University, Seoul, South Korea; UCL Institute of Child Health, UNITED KINGDOM

## Abstract

Dapagliflozin, a new type of drug used to treat diabetes mellitus (DM), is a sodium/glucose cotransporter 2 (SGLT2) inhibitor. Although some studies showed that SGLT2 inhibition attenuated reactive oxygen generation in diabetic kidney the role of SGLT2 inhibition is unknown. We evaluated whether SLT2 inhibition has renoprotective effects in ischemia-reperfusion (IR) models. We evaluated whether dapagliflozin reduces renal damage in IR mice model. In addition, hypoxic HK2 cells were treated with or without SGLT2 inhibitor to investigate cell survival, the apoptosis signal pathway, and the induction of hypoxia-inducible factor 1 (HIF1) and associated proteins. Dapagliflozin improved renal function. Dapagliflozin reduced renal expression of Bax, renal tubule injury and TUNEL-positive cells and increased renal expression of HIF1 in IR-injured mice. HIF1 inhibition by albendazole negated the renoprotective effects of dapagliflozin treatment in IR-injured mice. In vitro, dapagliflozin increased the expression of HIF1, AMP-activated protein kinase (AMPK), and ERK and increased cell survival of hypoxic HK2 cells in a dose-dependent manner. In conclusion, dapagliflozin attenuates renal IR injury. HIF1 induction by dapagliflozin may play a role in renoprotection against renal IR injury.

## Introduction

Although there are numerous causes of AKI including ureteral obstruction, calcineurin inhibitor toxicity and kidney ischemia, renal ischemic-reperfusion (IR)-induced injury is a major cause of AKI in patients undergoing renal transplantation [1–3]. Complex mechanisms are involved in IR injury, including hypoxic injury, tubular cell secretion of cytokines associated with apoptotic cell death, and acute inflammatory processes [4, 5]. Moreover, reperfusion following by ischemia generates massive quantities of reactive oxygen species (ROS), which result in tubular cell death [5]. However, ischemic tubule cells activate an adaptive process for survival against hypoxia. Hypoxia-inducible factor 1 (HIF1) is a key protein that regulates such adaptive cellular or tissue responses to hypoxia [6]. HIF1 has an oxygen-sensitive α subunit and a constitutively expressed β subunit [7]. Transcriptional regulation of HIF1 occurs when the HIF1 heterodimer binds to hypoxia response elements [7].

HIF1 functions as a protective molecule in hypoxic organs including the brain, heart, liver and kidney [8–11]. HIF1 induction by ischemic preconditioning reduced renal apoptosis and inflammation in IR injury [12]. In addition, stabilization of HIF1 by inhibition of prolyl hydroxylase domain (PHD) attenuated ischemic kidney injury [13].

Besides hypoxia, several other mediators can regulate HIF1, such as reactive oxygen species (ROS), cobalt chloride, nitric oxide, tumor necrosis factor-α, and angiotensin II [7]. HIF1 is involved in the regulation of many biological processes related to kidney function, including glucose and energy metabolism, angiogenesis, erythropoiesis, iron homeostasis, cell migration, vasomotor regulation, and cell–cell/cell–matrix interactions [7]. HIF1 induction reduces SGLT2 levels in kidney tubule cells; however, it is unclear whether SLGT2 inhibition regulates HIF1 [14].

Dapagliflozin, a new pharmacological therapy for type 2 diabetes, inhibits sodium/glucose cotransporter 2 (SGLT2), which results in excretion of glucose into the urine. SGLT2 inhibition reduced hyperfiltration, tubular oxidative stress, and oxygen consumption in a diabetic kidney [15–17]. However, SGLT2 inhibition also showed mild increase of oxygen consumption, which may induce renal hypoxia in non-diabetic rat kidney [16]. In this study, we evaluated whether dapagliflozin has a renoprotective effect in renal IR-injured mice and investigated the mechanism involved, including HIF1 regulation.

## Materials and Methods

### Mice and drugs

All of the experiments were performed using 10-week-old male C57BL/6 mice weighing 30–33 g each (Damul Science, Daejeon, Korea). The mice were given a standard laboratory diet (Damul Science, Daejeon, Korea) and water, and were cared for according to a protocol approved by the Institutional Animal Care and Use Committee of the Catholic University Medical School (CMCDJ-2014-001). The mice were divided into five groups: vehicle (Vh)-treated sham (n = 5), dapagliflozin-treated sham (n = 5), Vh-treated IR (n = 7), dapagliflozin-treated IR (n = 7), and albendazole and dapagliflozin treated IR (n = 7). Dapagliflozin (Astrazeneca Co., NJ, USA) was administrated via oral gavage at a dose of 10 mg/kg/day for 2 days, starting 24 h before surgery. Albendazole was injected subcutaneously 1 h before IR surgery.

IR injury was performed as described previously [5]. Briefly, mice were anesthetized with an intraperitoneal injection of ketamine (60 mg/kg body mass) and xylazine (8 mg/kg). After an abdominal incision, both renal pedicles were bluntly clamped. During the procedure, the mouse’s body temperature was kept constant at 35–36°C on a heating pad. The clamps were removed after 27 min of ischemia. Sham-treated control mice underwent a similar surgical procedure without clamping. Mice were sacrificed at 24 h after the surgical procedure, and the blood and kidneys were collected.

### Blood and tissue preparation

Blood was collected from the inferior vena cava at sacrifice under anesthesia. The blood was placed into pre-chilled Eppendorf tubes (4°C). Serum was separated by centrifugation for 10 min at 4°C. Aliquots of serum were shock-frozen using liquid nitrogen and stored at −70°C. Tissues were prepared as described previously [18]. Briefly, the left kidney was excised immediately after sacrifice and cut into three coronal sections. Two pieces of the kidney were snap-frozen in liquid nitrogen and kept at −70°C for subsequent RNA extraction and protein analysis. The other kidney portion was fixed in 10% buffered formaldehyde at room temperature and then embedded in paraplast (Sherwood Medical, St. Louis, MO, USA) for light microscopy and terminal deoxynucleotidyl transferase dUTP nick end labeling (TUNEL) analyses.

### Cell culture and drug treatment

HK-2, an immortalized proximal tubule epithelial cell line, was purchased from the American Type Culture Collection (Manassas, VA, USA) and cultured. Briefly, cells were passaged every 3–4 days in 100-mm dishes (Falcon, Bedford, MA, USA) using Dulbecco’s modified Eagle’s medium-F12 (Sigma-Aldrich, St. Luis, MO, USA) supplemented with 10% fetal bovine serum (Life Technologies Inc., Gaithersburg, MD, USA), insulin-transferrin-sodium selenite media supplement (Sigma-Aldrich), 100 U/ml penicillin, and 100 mg/mL streptomycin (Sigma-Aldrich). For experimental use, these cells were incubated in a humidified atmosphere of 5% CO2, 95% air at 37°C for 24 h and subcultured at 70–80% confluence.

Hypoxia was simulated by immersing HK-2 cell monolayers in mineral oil (Sigma-Aldrich) for 30 min at 37°C. After washing extensively with PBS, the cells were incubated in DMEM/F12 medium supplemented with 10% FBS for 24 h after medium replacement. These cells were pretreated with various doses of dapagliflozin for 2hr. The cell were harvested at the end of hypoxia for molecular analysis including HIF-1, AMPK, and EKR. For glucose assay, glucose in the medium was measured with glucose assay kit (Abcam ab65333), according to the manufacturer’s instructions. Glucose consumptions in each groups (control: normoxia and non-treated, Dapa1: normoxia and dapagliflozin 1μM treated, Dapa10: normoxia and dapagliflozin 10μM treated, Hypoxia: hypoxia with non-treated, Hypoxia Dapa1: hypoxia with dapagliflozin 1μM treated, Hypoxia Dapa10: hypoxia with dapagliflozin 10μM treated) were calculated, comparing to control group. All cellular experiment **repeated three times** for western blot and glucose assay.

### Cell viability assay

To perform the cell survival assay, cells were collected after 24 h incubation with vehicle or dapagliflozin pretreatment in 30-min ischemia and surviving cells were counted with Trypan blue staining. The percentage survival was determined by quantization of the relative viable number of treated cells divided by the viable number of untreated cells.

### Western blot

Western blot was performed for protein analysis as described previously [4]. Briefly, kidney sections were homogenized in protein extraction solution (PRO-PREP, iNtRON, Sungnam-si, Korea); 40 μg of total protein were loaded in a stacking polyacrylamide gel and resolved on an 8 and 15% polyacrylamide gel with a biotinylated molecular weight standard marker. Then, the samples were wet-transferred to a 0.2-μm nitrocellulose membrane (Amersham Pharmacia biotech, NY, USA). The blots were blocked for 1 h with 5% non-fat dry milk in TBST buffer (20 mM Tris–HCl [pH 7.6], 0.8% NaCl and 0.05% Tween 20) and incubated overnight at 4°C with a 1:1000 anti-HIF-1-alpha antibody (HIF-1; Abcam, Cambridge, MA, USA), AMP-activated protein kinase (AMPK; Cell Signaling Technology, Inc., Beverly, MA, USA), anti-Bax antibody (Cell Signaling Technology, Inc.), anti-Bcl2 antibody (Cell Signaling Technology, Inc.), and poly ADP ribose polymerase (PARP, Cell Signaling Technology, Inc.). The blots were incubated with 1:1000 secondary anti-rabbit IgG-HRP-linked antibody or 1:1000 secondary anti-goat IgG- HRP-linked antibody (Cell Signaling Technology, Inc.) for 1 h. The bands were detected using enhanced chemiluminescence (Millipore, Billerica, MA, USA), and exposed to films. The optical density for quantification was obtained using Gel-Pro Analyzer version 3.1 (Media Cybernetics, Silver Spring, MD, USA).

### Tubular injury score

Paraffin-embedded kidney pieces were cut into 4-μm sections and mounted on glass slides. The sections were deparaffinized with xylene, stained with hematoxylin and eosin (H&E) and periodic acid-Schiff (PAS), and examined under an Olympus BX51 microscope (Olympus, Tokyo, Japan). In the H&E sections, renal cortical vacuolization, peritubular/proximal tubule leukocyte infiltration, and proximal tubule simplification were evaluated and scored as follows: 0, normal; 1, mild injury; 2, moderate injury; 3, severe injury. Tubular damage (epithelial necrosis) in PAS-stained sections was scored as follows: 0, normal; 1, <10%; 2, 10–25%; 3, 26–75%; 4, >75%. Tubular necrosis was defined as the loss of proximal tubular brush border blebbing of apical membranes, or intraluminal aggregation of cells and proteins, as described previously [4]. Kidney sections were evaluated by an experienced pathologist in a blind fashion. At least 5 fields (magnification, ×200) were reviewed for each slide (n = 5, each groups).

### TUNEL assay

TUNEL was performed using the *in situ* Apoptosis Detection Kit (S7100-KIT; EMD milipore, CA, USA) as described previously [19]. Briefly, the 4-μm-thick paraffin embedded sections were dewaxed. The sections were incubated in 0.3% H_2_O_2_ at room temperature to eliminate the endogenous peroxidase activity. Proteinase K (10 μg/mL in 0.1-M Tris, 50-mM EDTA [pH 8]) was applied to the sections for 15 min at room temperature. TdT enzyme was applied to the sections and incubated in a humidified chamber for 1 h at 37°C to allow extension of the nicked ends of the DNA fragments with digoxigenin-dUTP. Color was developed using 0.05% DAB with 0.006% H_2_O_2_ as substrate. For negative controls, distilled water was used instead of TdT enzyme.

### Immunohistochemistry

Immunohistochemistry was performed as described previously [17]. Paraffin wax-embedded tissues were cut into 4 μm sections, mounted on glass slides, and stained using indirect immunoperoxidase. The slides were processed for identification of HIF-1-alpha expression (anti-HIF-1-alpha; Abcam, Cambridge, MA, USA), followed by detection using diaminobenzidine (Sigma Chemical Co., St. Louis, MO, USA). All of the samples were evaluated under an Olympus BX51 microscope. The the areas stained for HIF1-alpha, as percentages of the total area in 10 different fields of each section under 200× magnification, were determined automatically using a digital camera-based image analyzer (Metamorpho, ver. 4.6).

### Statistical analysis

Data are reported as means ± standard deviation (SD). Multiple comparisons among groups were performed using one-way analysis of variance (ANOVA) with a post-hoc Bonferroni test correction (SPSS 11.0 for Windows; SPSS, Inc., Chicago, IL, USA). *P* values < 0.05 were considered to indicate statistical significance.

## Results

### The effects of dapagliflozin on renal function and histology

**Fig 1:**
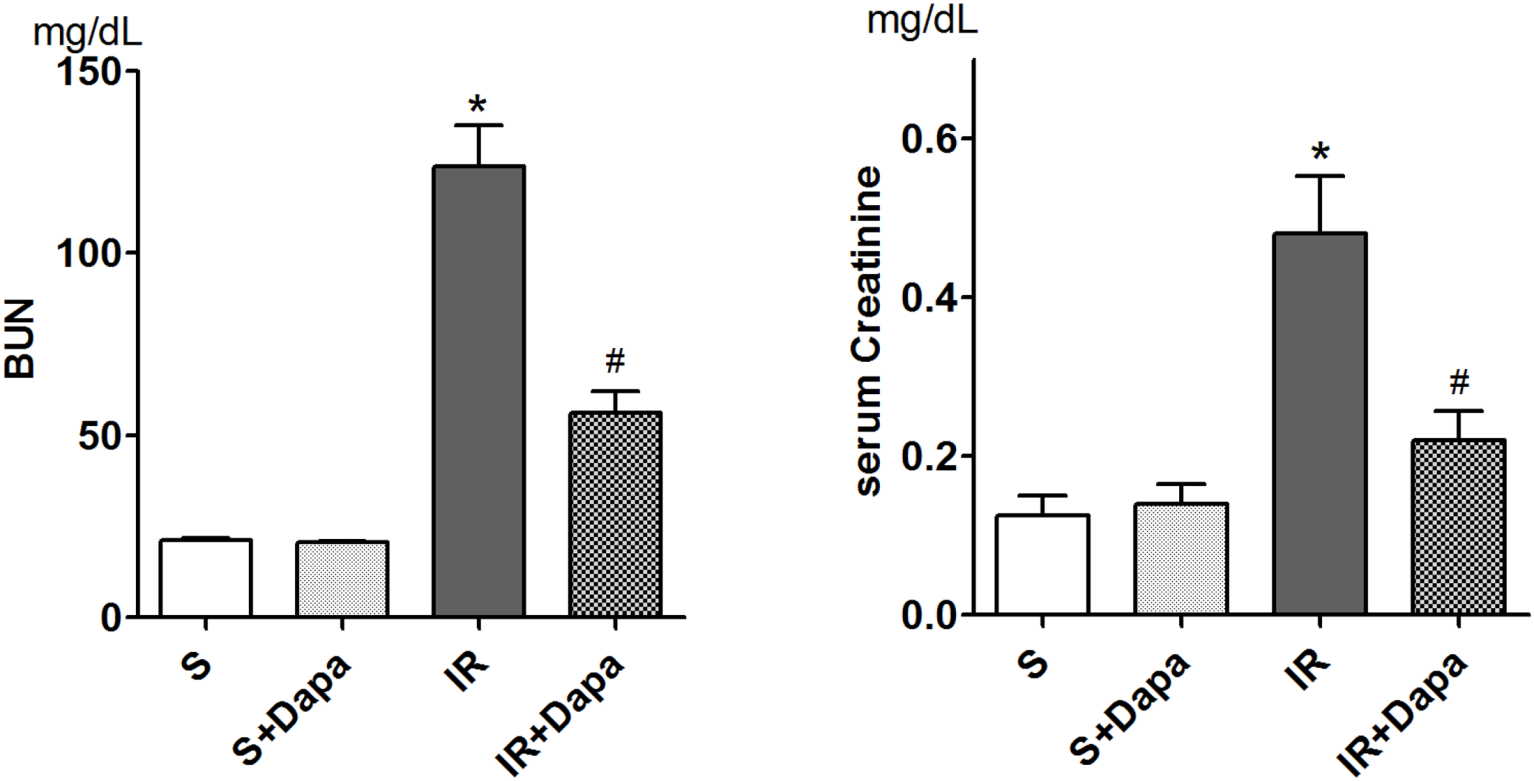
Effects of dapagliflozin on renal function (S(n = 5), sham; S + Dapa (n = 5), sham + dapagliflozin; IR(n = 7), vehicle-treated renal IR mice; IR + Dapa (n = 7), dapagliflozin-treated IR mice). Dapagliflozin pretreatment reduced BUN and serum creatinine in IR-injured mice. *P < 0.05 vs. vehicle-treated sham, #*P <* 0.05 vs. vehicle-treated IR. Bar represents mean ± s.d.(Standard deviation).

**Fig 2:**
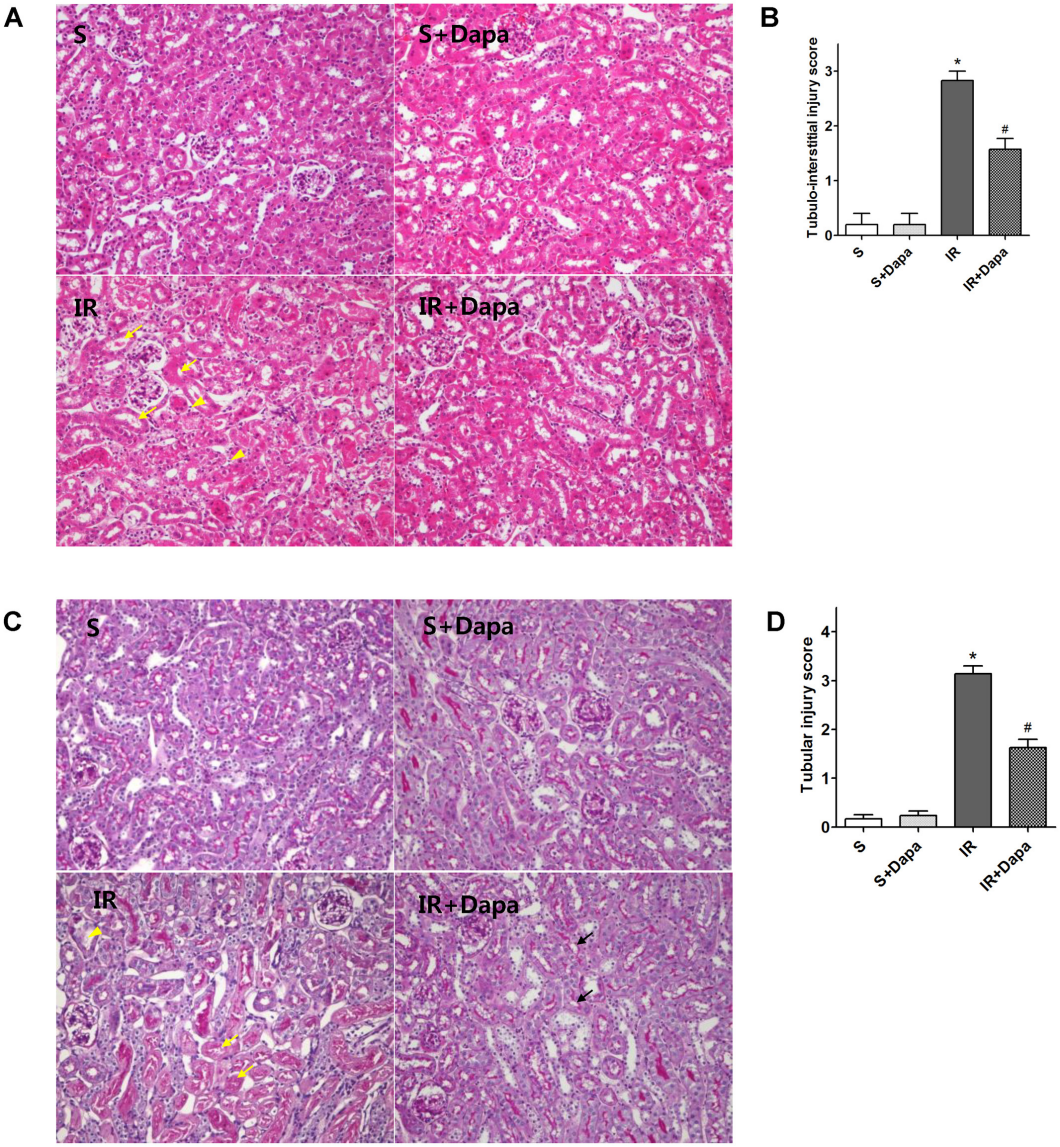
Representative kidney section stained for hematoxylin and eosin (H&E) and periodic acid-Schiff (PAS). (S(n = 5); S + Dapa (n = 5), IR(n = 7), IR + Dapa (n = 7)). (A) H&E stain, yellow arrows indicate cell debri and tubular necrosis. Yellow arrowheads indicate inflammatory cells. Original magnification, 200×. (B) Semi-quantitative analysis of tubule interstitial injury in wild-type and dapagliflozin- and/or albendazole-treated mice 24 h after renal IR injury (C) PAS stain. Yellow arrows indicate necrotized tubules or cast formation. Yellow arrowheads indicate loss of brush border or dilated tubules. Black arrow indicates brush border. Original magnification, 200×. (D) Semi-quantitative analysis of tubular injury in wild-type and dapagliflozin- and/or albendazole-treated mice 24 h after renal IR injury. *P < 0.05 vs. vehicle-treated sham, #*P <* 0.05 vs. vehicle-treated IR. Bar represents mean ± s.d.

The levels of BUN and serum creatinine in mice were significantly elevated 24 h after IR injury. Dapagliflozin reduced the level of BUN and serum creatinine in IR-injured mice (Fig 1). In histologic evaluation, IR-injured kidneys showed loss of brush border, vacuolization, and desquamation of epithelial cells in renal tubular epithelium. Pretreatment with dapagliflozin attenuated the renal tubular injuries (Fig 2).

### Anti-apoptotic effect of dapagliflozin

**Fig 3:**
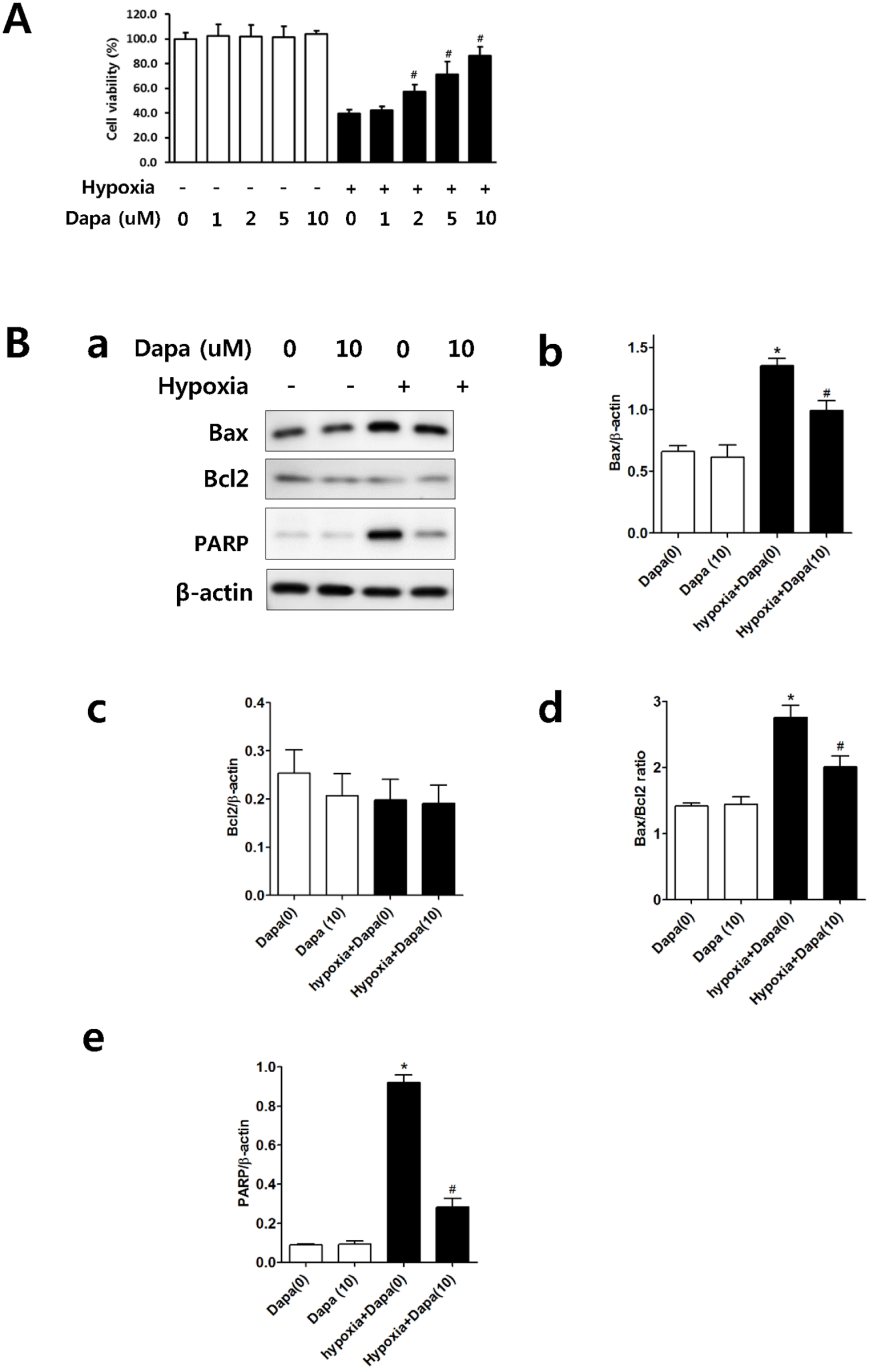
Effects of dapagliflozin on HK2 cell survival and apoptosis. (A) Dapagliflozin significantly increased the cell survival in hypoxic HK2 cell in a dose-dependent manner. (B) Representative western blot (B-a). Dapagliflozin significantly decreased the Bax expression and Bax/Bcl2 ratio in hypoxic HK2 cells (B-b and d). However, Bcl2 expression was unaffected by dapagliflozin in hypoxic HK2 cells (B-c). Dapagliflozin significantly decreased the PARP expression in hypoxic HK2 cells (B-e) *P < 0.05 vs. normoxic HK2 cells, #*P <* 0.05 vs. control hypoxic HK2 cells. Bar represents mean ± s.d. All cellular experiment **repeated three times for cell viability test and western blot.**

In vitro, dapagliflozin pretreatment showed no effect on the cell survival of normoxic HK2 cells. Hypoxia significantly decreased the cell viability of HK2 cells compared with control cells, and dapagliflozin pretreatment of hypoxic HK2 cells significantly improved the cell viability in a dose-dependent manner (Fig 3A). Bax expression was elevated significantly in hypoxic HK2 cells, whereas dapagliflozin pretreatment decreased Bax expression in hypoxic HK2 cells. The Bax/Bcl-2 ratio, used as an index of apoptotic signaling, showed a significant increase in hypoxic HK2 cells, whereas dapagliflozin pretreatment decreased the Bax/Bcl2 ratio (Fig 3B). Also, dapagliflozin pretreatment decreased PARP expression in hypoxic HK2 cells.

**Fig 4:**
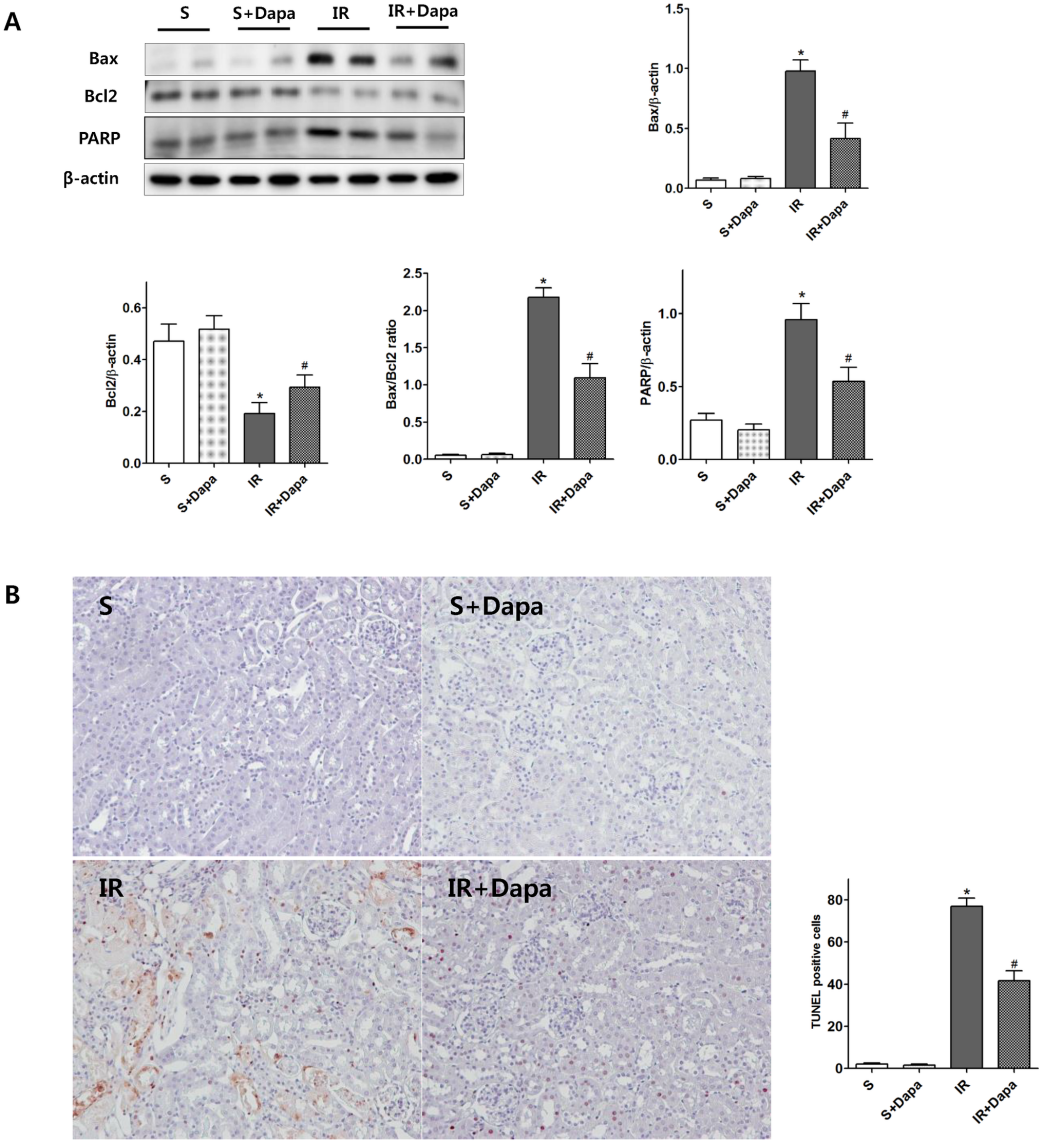
Effects of dapagliflozin on apoptosis in IR-injured kidneys (S(n = 5); S + Dapa (n = 5), IR(n = 7), IR + Dapa (n = 7)). (A) Western blot analysis shows dapagliflozin decreased Bax expression and increased Bcl2 expression in IR-injured kidneys. Dapalgliflozin decreased PARP expression in IR-injured kidneys. (B) Representative kidney section. Dapagliflozin decreased the TUNEL-positive cells in an IR-injured kidney. *P < 0.05 vs. sham kidney, #*P <* 0.05 vs. IR kidney. Bar represents mean ± s.d.

In vivo, Bax expression was increased significantly and Bcl2 expression was decreased in IR-injured kidneys compared with the expression levels in sham-treated mice. However, dapagliflozin pretreatment decreased Bax expression and increased Bcl2 expression in IR-injured kidneys. In addition, dapagliflozin pretreatment decreased PARP expression in IR injured kidneys (Fig 4A). The TUNEL assay showed a significant increase in TUNEL-positive cells in IR-injured kidneys; however, dapagliflozin pretreatment significantly reduced the number of TUNEL-positive cells in IR-injured kidneys (Fig 4B).

### The effects of SGLT2 on HIF1

**Fig 5:**
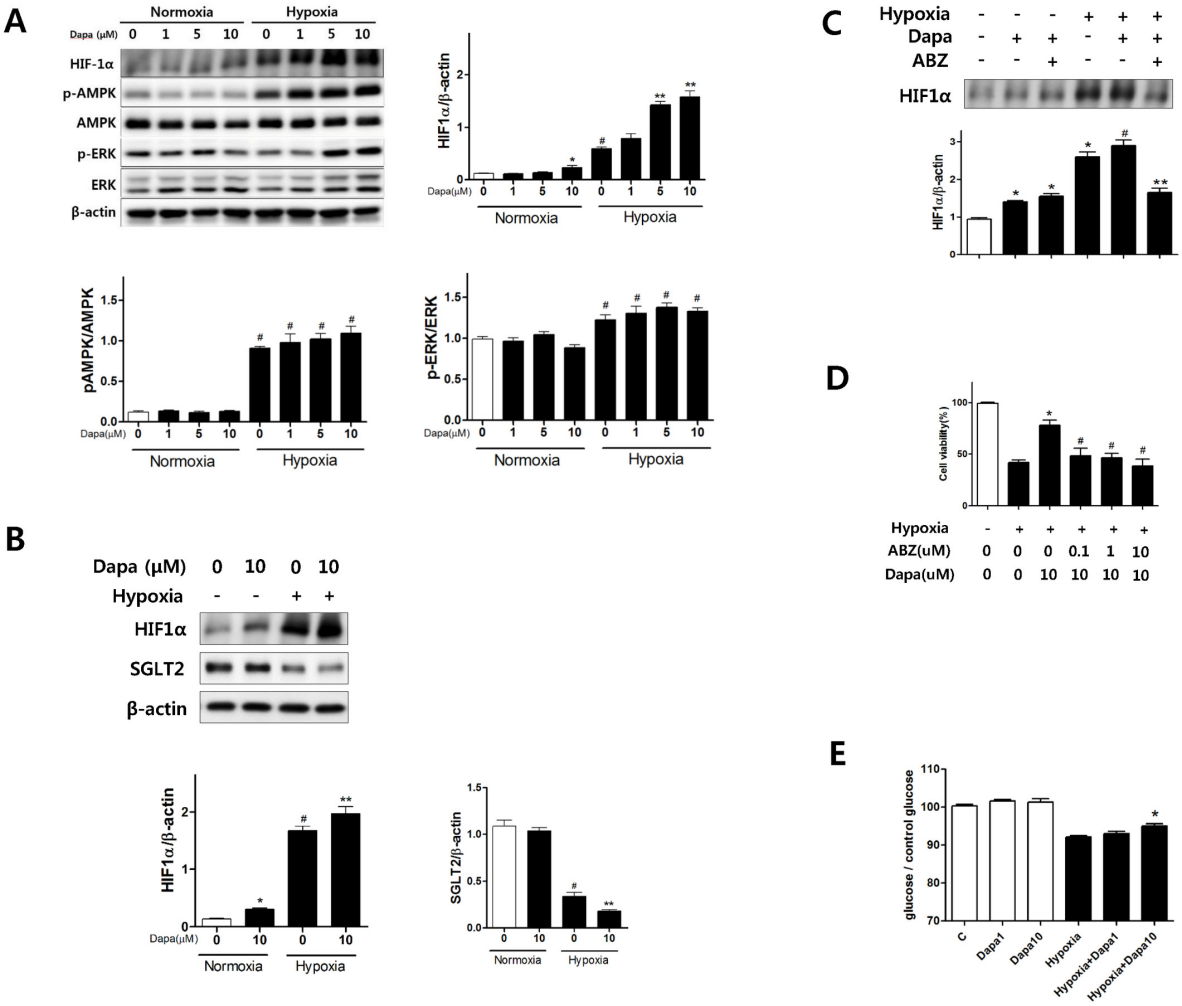
Effects of dapagliflozin on HIF1 in HK2 cells. (A) Representative Western blot: dapagliflozin increased HIF1 expression and phosphorylation of AMPK and ERK in hypoxic HK2 cells. *P < 0.05 vs., dapagliflozin nontreated normoxic HK2 cells, #*P <* 0.05 vs. normoxic HK2 cells. **P < 0.05 vs. control hypoxic HK2 cells. (B) Representative Western blot: hypoxia increased HIF1 expression and decreased SGTL2 expression. Dapagliflozin pretreatment decrease SGLT2 expression in hypoxic HK2 cells. *P < 0.05 vs., dapagliflozin nontreated normoxic HK2 cells, #*P <* 0.05 vs. normoxic HK2 cells. **P < 0.05 vs. control hypoxic HK2 cells. (C) Dapaglilfozin pretreatment increase HIF1 expression in normoxic and hypoxic HK2 cells compared to control and hypoxic control HK2 cells respectively. Albendazole decreased HIF1 expression in dapagliflozin-treated hypoxic HK2 cells. *P < 0.05 vs., control HK2 cells, #*P <* 0.05 vs.control hypoxic HK2 cells. **P < 0.05 vs. albendazole nontreated—dapagliflozin treated hypoxic HK2 cells. (D) Albendazole decreased cell survival in dapagliflozin-treated hypoxic HK2 cells. *P < 0.05 vs., control hypoxic HK2 cells #*P <* 0.05 vs.dapagliflozin treated hypoxic HK2 cells. (E) Dapagliflozin decreased glucose uptake in hypoxic HK2 cells. *P < 0.05 vs., hypoxic HK2 cells. Bar represents mean ± s.d. All cellular experiment **repeated three times for western blot, cell viability test, and glucose uptake test.**

Hypoxic HK2 cells showed marked elevation in HIF1 expression compared with that in control HK2 cells. Dapagliflozin pretreatment further increased the HIF1 expression in hypoxic HK2 cells. Additionally, dapagliflozin increased AMPK and EKR phosphorylation in hypoxic HK2 cells. Although a high dose of dapagliflozin elevated HIF1 expression, it showed no effect on the phosphorylation of AMPK and ERK in normoxic HK2 cells (Fig 5A). Hypoxia significantly decreased the SGLT2 expression in HK2 cells. Dapagliflozin pretreatment significantly elevated HIF1 expression and decreased SGLT2 expression (Fig 5B). HIF1 inhibitor, albendazole pretreatment significantly reduced the dapagliflozin-induced HIF elevation (Fig 5C). In addition, albendazole pretreatment reduced the dapagliflozin-induced cell viability improvement (Fig 5D). 10μM of dapagliflozin treated hypoxic HK2 cell group showed elevated glucose concentration of culture medica, compared to non-treated hypoxic HK2 cell group. However, in normoxic condition, there were no significant difference between dapagliflozin treated groups and non-treated group (Fig 5E).

**Fig 6:**
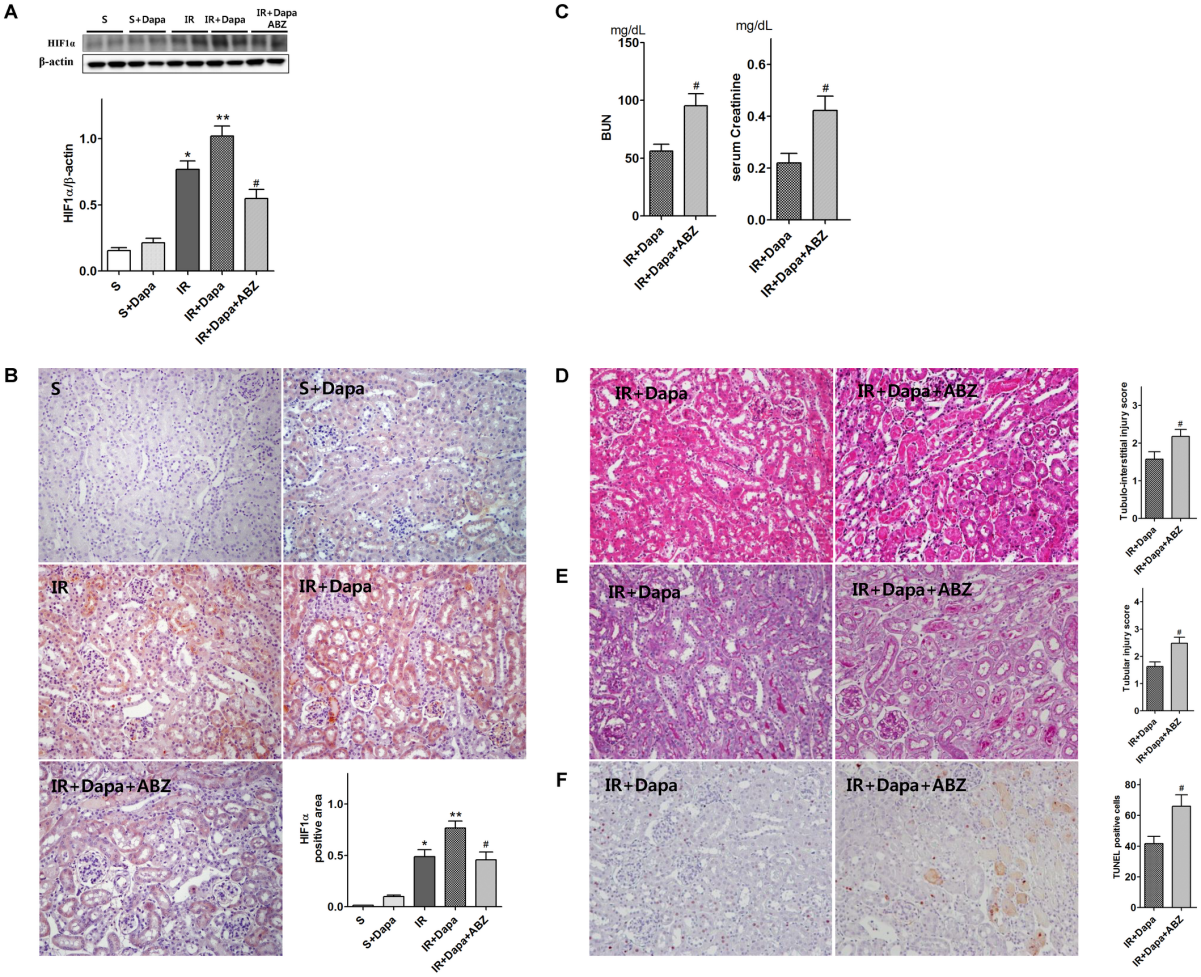
Effects of dapagliflozin on HIF1 in IR-injured kidneys (S (n = 5), sham; S+Dapa (n = 5), sham+dapagliflozin; IR (n = 7); vehicle-treated renal IR mice; IR+Dapa (n = 7), dapagliflozin-treated IR mice; IR+Dapa+ABZ (n = 7), albendazole- and dapagliflozin-treated IR mice). (A) Representative Western blot: dapagliflozin increased HIF1 expression in IR-injured kidneys. Albendazole decreased HIF1 expression in dapagliflozin-treated IR-injured kidney. (B) Representative Immunohistochemistry of HIF1. Dapagliflozin increase the HIF1 stained area in tubular area of IR kidney. Original magnification, 200× (C) The effects of dapagliflozin on renal function. Albendazole treatment elevated BUN and serum creatinine in dapagliflozin-treated IR mice. (D–F) Representative kidney section: albendazole treatment increase tubulointerstitial injury and TUNEL-positive cells in IR-injured kidneys. *P < 0.05 vs. vehicle-treated sham, **P < 0.05 vs. vehicle-treated IR, #*P <* 0.05 vs. dapagliflozin-treated IR. Bar represents mean ± s.d.

In mice, IR-injured kidneys showed marked elevation in HIF1 expression. Moreover, dapagliflozin pretreatment significantly elevated the HIF1 expression in IR-injured renal tubular cells (Fig 6A and 6B). However, albendazole treatment reduced the HIF1 expression (Fig 6A and 6B). Also, albendazole treatment significantly increased the BUN and serum creatinine levels (Fig 6C), and increased the tissue injury and TUNEL-positive cells in dapagliflozin-treated IR-injured mice (Fig 6D–6F).

## Discussion

This study clearly demonstrated that dapagliflozin, an SGLT2 inhibitor, improves renal function and reduces apoptotic cell death in IR-injured kidneys in mice. In addition, dapagliflozin induces HIF1 and reduces the Bax/Bcl2 ratio in ischemic renal tissue and cultured ischemic tubular cells.

HIF1 plays a beneficial role in cellular adaptation to IR-induced injury [20, 21]. Although several reports have found no protective effect of HIF1 in liver IR injury [22, 23], many studies have shown that genetic or pharmacologic activation of HIF1 ameliorates IR injury in various organs [8–11]. In kidney, IR-injured HIF1*α*-KO mice showed more severe renal tubulointerstitial injury and deteriorated renal function compared with wild-type IR-injured mice [24]. HIF1 preservation via prolyl hydroxylase domain (PHD) inhibition showed a potent renal protective effect in IR-injured rats [25]. Moreover, HIF1 induction ameliorates apoptosis in IR-injured kidney via Bax and/or Bcl2 regulation [25, 26]. In our study, dapagliflozin increased HIF1 expression, reduced Bax expression and TUNEL positive cells, and increased Bcl2 expression in IR-injured kidneys. Moreover, HIF1 inhibition by albendazole ameliorated the dapagliflozin-induced benefits on IR-injured kidneys. These results suggest that the renoprotective effects of dapagliflozin are associated with the induction of HIF1.

Dapagliflozin is a newly developed diabetes medicine. Because the SGLT2 in the proximal tubule accounts for the vast majority of glucose reabsorption by the kidney, inhibition of SGLT2 can decrease glucose levels in the blood. Nakamura et al. reported that high glucose or advanced glycation end-product (AGE) did not elevate SLGT2 expression in human proximal tubular cells [27]. However, recent studies have shown that the effects of specific SGLT2 inhibition in human proximal tubular (HK2) cells exposed to high glucose reduced inflammatory and fibrotic markers in both polarized and non-polarized settings [15, 28]. It is unclear whether SGLT2 plays a role in proximal tubular cells under normal glucose conditions. Zapata-Morales et al. reported that hypoxic conditions decrease SLGT2 expression in porcine proximal tubular cells [14]. Nakamura et al. reported that insulin and ROS increase the expression of SGLT2 and glucose uptake in proximal tubular cells [27]. Therefore, SGLT2 may play a role in various tubular injuries.

Zapata-Morales et al. reported that HIF1 induction by hypoxia or cobalt chloride suppressed the expression of SGLT2 in proximal tubular cells [14]. Similarly, our study showed that hypoxic insult increased HIF1 expression and decreased SGLT2 expression in HK2 cells. It is unknown whether SLGT2 regulates HIF1. SGLT2 inhibition by dapagliflozin increased the expression of HIF1 in ischemic HK2 cells; however, this study did not address whether overexpression of SLGT2 regulates HIF1 expression in HK2 cells.

Although several factors are known to regulate HIF, the mechanism underlying dapagliflozin-induced HIF1 expression in hypoxic or normal HK2 cells is unclear. Phosphorylation of AMPK and EKR induces HIF1 expression in hypoxic conditions [29, 30]. In addition, PHDs are inhibited during hypoxia. Decreased PHDs lead to the accumulation of HIF1*α subunit*, which dimerize with HIF1*β* resulting in increased HIF1 levels. Activation of the PI3K/Akt signaling pathway plays a major role in HIF*α* accumulation during the reperfusion period [31]. In our study, dapagliflozin elevated phosphorylated AMPK and EKR in hypoxic HK2 cells. It is possible that elevated phosphorylated AMPK and ERK induce HIF1. In addition, dapagliflozin treatment showed decreased glucose uptake and reduced SGLT2 expression in hypoxic HK2 cells. Decreased glucose uptake in hypoxic cells may induce AMPK and EKR phosphorylation. Although this study did not address whether SGLT2 regulates HIF1 directly, dapagliflozin-induced cellular glucose deficiency may induce HIF1. However, in normoxic conditions, dapagliflozin did not affect the glucose consumption in tubular cells. Moreover, dapagliflozin did not increase the phosphorylation of AMPK and ERK. HIF1 expression increased in dapagliflozin-treated control HK2 cells, suggesting that dapagliflozin regulates HIF1 directly, and not via low cellular glucose. Layton et at reported that acute SGLT2 inhibition increase 5% oxygen consumption (calculated by ATP consumption) in renal cortex from baseline non-diabetic kidney [16]. Although it is not evaluated IR injured kidney, it may be possible that SGLT2 inhibition induced increase of APT consumption, result in HIF-1 generation. Recently, SGLT2 inhibition reduced cardiovascular mortality in type 2 diabetes patients [32]. It could be possible that SLGT2 inhibitor may favorable effect to damaged heart via HIF1 activation. However, It is uncertain not only whether long term treatment of dapagliflozin may affect the HIF1 expression, but also whether dapagliflozin may affect the damaged heart. Although HIF1 activation reduce various acute organ injury, long term HIF1 activation may induce tissue fibrosis.

This study showed that dapagliflozin attenuates renal IR injury. Elevated HIF1 expression by dapagliflozin pretreatment may play a major role in the protection of IR-injured renal tubule cells.
