# Correction: Dapagliflozin, SGLT2 Inhibitor, Attenuates Renal Ischemia-Reperfusion Injury

**DOI:** 10.1371/journal.pone.0160478

**Published:** 2016-07-28

**Authors:** Yoon-Kyung Chang, Hyunsu Choi, Jin Young Jeong, Ki-Ryang Na, Kang Wook Lee, Beom Jin Lim, Dae Eun Choi

The affiliation for the sixth author, Beom Jin Lim, is spelled incorrectly. The correct affiliation is: Department of Pathology, College of Medicine, Yonsei University, Seoul, South Korea.
